# Flexible PVC materials grafted with castor oil derivative containing synergistic flame retardant groups of nitrogen and phosphorus

**DOI:** 10.1038/s41598-018-38407-4

**Published:** 2019-02-11

**Authors:** Puyou Jia, Yufeng Ma, Meng Zhang, Lihong Hu, Qiaoguang Li, Xiaohui Yang, Yonghong Zhou

**Affiliations:** 1 Institute of Chemical Industry of Forest Products, Chinese Academy of Forestry (CAF), Co-Innovation Center of Efficient Processing and Utilization of Forest Resources, Nanjing Forestry University and Key Lab of Biomass Energy and Materials, Jiangsu Province, 16 Suojin North Road, Nanjing, 210042 P.R. China; 2 Nanjing Forestry University, College of Materials Science and Engineering, 159 Longpan Road, Nanjing, 210037 P.R. China; 3 School of Chemistry and Chemical Engineering, Zhongkai University of Agriculture and Engineering, Guangzhou, 510225 P.R. China

## Abstract

Internally plasticized PVC by replacement of chlorine with castor oil derivative containing synergistic flame retardant groups of nitrogen and phosphorus, that is, castor oil derivative grafted onto PVC matrix, is reported. Low glass transition temperature (Tg) of modified PVC was produced although thermal stability was reduced. However, the migration was completely suppressed. The combination of castor oil derivative containing synergistic flame retardant groups of nitrogen and phosphorus with PVC matrix through modifying PVC materials with click reaction prepared flexible PVC materials with zero migration and enhanced flame retardant property.

## Introduction

Plasticizer is an important plastic additive, which has been widely used around the world. It plays an essential role in many types of plastic products. As the main plasticizer in plastic industry, phthalate esters account for large parts of the additives. The truth is that plasticizer industry is a large scale chemical industry taking phthalate esters as center based on petrochemical resources^1^. Phthalate esters are easily extracted by solvent. The loss of plasticizer will reduce performance of PVC products and properties of the PVC products will change with increasing time^2,3^. In addition, flammable nature of phthalate esters further limits its application in plastic products with high fire protection requirements. Almost all of phthalate esters derive from petrochemical resources, the industrial model is not sustainable with the depletion of fossil resources. To solve these problems, many alternatives to phthalate plasticizers have been studied. Bio-based plasticizers have been developed or used commercially such as cardanol-based plasticizer^4^, epoxidized safflower oil^5^, glycerol esters^6^, and triethyl citrate^7^. In order to improve flame retardancy of bio-based plasticizer, a series of vegetable oil based phosphate plasticizer have been synthesized in the previous studies^8–12^. Castor oil is an important chemical raw material, because its hydroxylated fatty acid structure can be used to prepare different chemical products^8^. Vegetable oil based additive containing phosphorus groups^9,10^, phenanthrene ring groups^11^ and 1,3,5-Tris (2-hydroxyethyl) cyanuric acid (THEIC) groups^12^ were synthesized and used as plasticizers for preparing flame retardant PVC materials respectively. However, all of these plasticizers are easily leached. The use of internal plasticizers can avoid migration of plasticizer from PVC products. Internal plasticizers are connected to the chemical structure of PVC matrix by chemical modification, because they are the inherent parts of the PVC chains^13^. “Click chemistry” is an efficient chemical synthesis method to prepare internally plasticized PVC materials^14,15^. It has been paid many attentions for the preparation of internally plasticized PVC materials^16,17^.

Based on the above conclusion, the application of castor oil based flame retardant plasticizer grafting onto PVC matrix would suppress migration and improve its flame retardancy. We now report a method to prepare PVC materials with zero migration using castor oil derivative containing synergistic flame retardant groups of nitrogen and phosphorus. The new flexible PVC materials would have potential application value in making flame retardant PVC materials without migration.

## Results

### Structure of propargyl ether THEIC-MR-P

**Figure 1:**
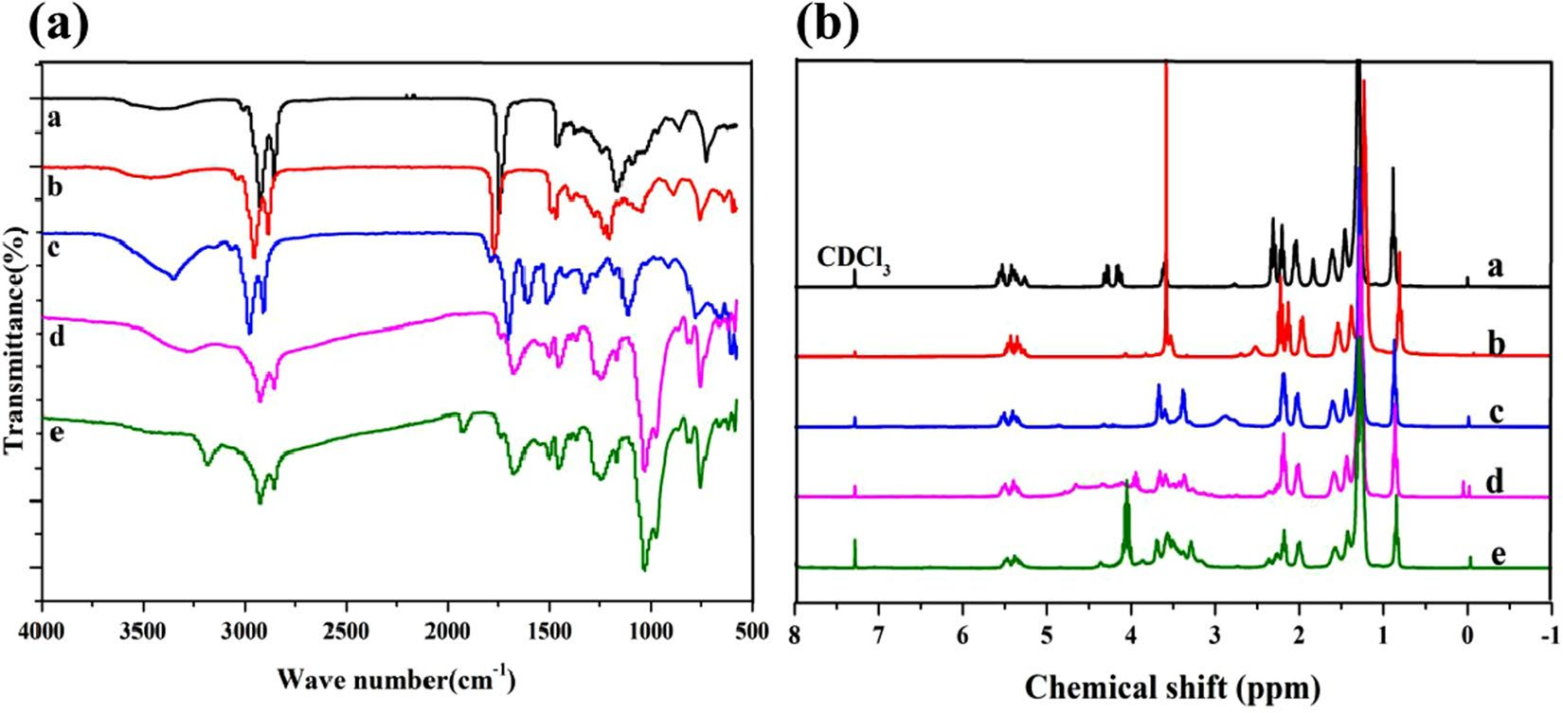
(**a**) FT-IR of castor oil (a), MR (b), MR-THEIC (c),THEIC-MR-P (d) and propargyl ether THEIC-MR-P (e). (**b**) ^1^H NMR of castor oil (a), MR (b), MR-THEIC (c),THEIC-MR-P (d) and propargyl ether THEIC-MR-P (e).

Chemical structure of propargyl ether THEIC-MR-P was detected by FT-IR and ^1^H NMR. Figure 1(a) shows the FT-IR of castor oil, castor oil derivatives and propargyl ether THEIC-MR-P. FT-IR and ^1^H NMR spectra of castor oil, MR and THEIC-MR were detected and discussed in detail in our previous studies^12,18^. The hydroxyl absorption peak of THEIC-MR-P showed weaker at around 3297 cm^−1^ than THEIC-MR in Fig. 1(a). The peak at around 1020 cm^−1^ was derived from P-O-C. The peak at around 970 cm^−1^ and 800 cm^−1^ was derived from P-C and P-O-CH_2_CH_3_^12,18^, which indicated that THEIC-MR-P was obtained. Hydroxyl absorption peak of THEIC-MR-P disappeared at around 3297 cm^−1^ in the FT-IR of propargyl ether THEIC-MR-P, new peaks appeared at around 2000 cm^−1^ and 3250 cm^−1^, which was attributed to C≡C stretch and alkyne C-H stretch respectively. The results showed that propargyl ether THEIC-MR-P was obtained. ^1^H NMR spectra of THEIC-MR-P was showed in Fig. 1(b), the peak at 0.7 ppm attributing to protons of -CH_3_ of THEIC-MR-P was stronger than THEIC-MR. The peak at 2.6 ppm was corresponded to protons of methylene deriving from THEIC-MR-P^12,18^. In addition, the peak at 3.6 ppm corresponding to the protons of the different -OH groups become weaker than THEIC-MR, all of the results indicated that THEIC-MR-P was prepared. One new peak at 4.1 ppm in the ^1^H NMR of propargyl ether THEIC-MR-P was corresponded to protons of -CH_2_-, which was connected to alkynyl groups, and another new peak at 2.4 ppm was attributed to protons of alkynyl groups compared with THEIC-MR-P, which illustrated that propargyl ether THEIC-MR-P was obtained.

### Structure of PVC materials

**Figure 2:**
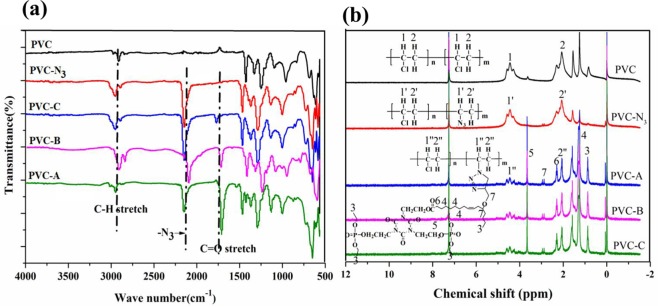
(**a**) FT-IR spectra of modified PVC; (**b**) ^1^H NMR spectra of modified PVC.

Figure 2 shows the FT-IR spectra of PVC and modified PVC materials. As seen from Fig. 2(a), new strong peak at around 2100 cm^−1^ in the FT-IR spectra of PVC-N_3_ was attributed to -N_3_ groups, which illustrated that PVC was converted to PVC-N_3_^16^. The peak of -N_3_ groups disappeared slowly with more propargyl ether THEIC-MR-P grafted onto the chains of azide functionalized PVC, while the FT-IR speatra of ester groups at around 1760 cm^−1^ enhanced. In addition, the peaks of P-O-C (1090 cmcm^−1^), P-C (960 cm^−1^) and P-O-CH_2_CH_3_ (930 cm^−1^) presented stronger with more propargyl ether THEIC-MR-P grafted onto chains of azide functionalized PVC^19,20^, which indicated that flexible PVC materials grafted with castor oil derivative containing synergistic flame retardant groups of nitrogen and phosphorus were obtained. Figure 2(b) shows the ^1^H NMR spectra. A single peak at 4.4 ppm was corresponded to the methylidyne protons, which was connected to -N_3_ and -Cl groups^19,20^. Furthermore, broad signals between at 2.4 ppm and 2.7 ppm were attributed to the methylene protons, which were connected to methylene group^19,20^. These results illustrated that castor oil derivative was attached onto PVC-N_3_ chains. ^1^H NMR spectra of modified PVC, multiple peaks at 1.0–1.2 ppm was corresponded to methyl protons, a single peak at 4.1 ppm was attributed to the methylene protons. The weak peaks at 5.5 ppm were corresponded to protons of olefin groups deriving from long chains of propargyl ether THEIC-MR-P. These data illustrated that propargyl ether THEIC-MR-P, as inherent parts of the polymer molecules, was grafted onto PVC-N_3_ chains.

**Figure 3:**
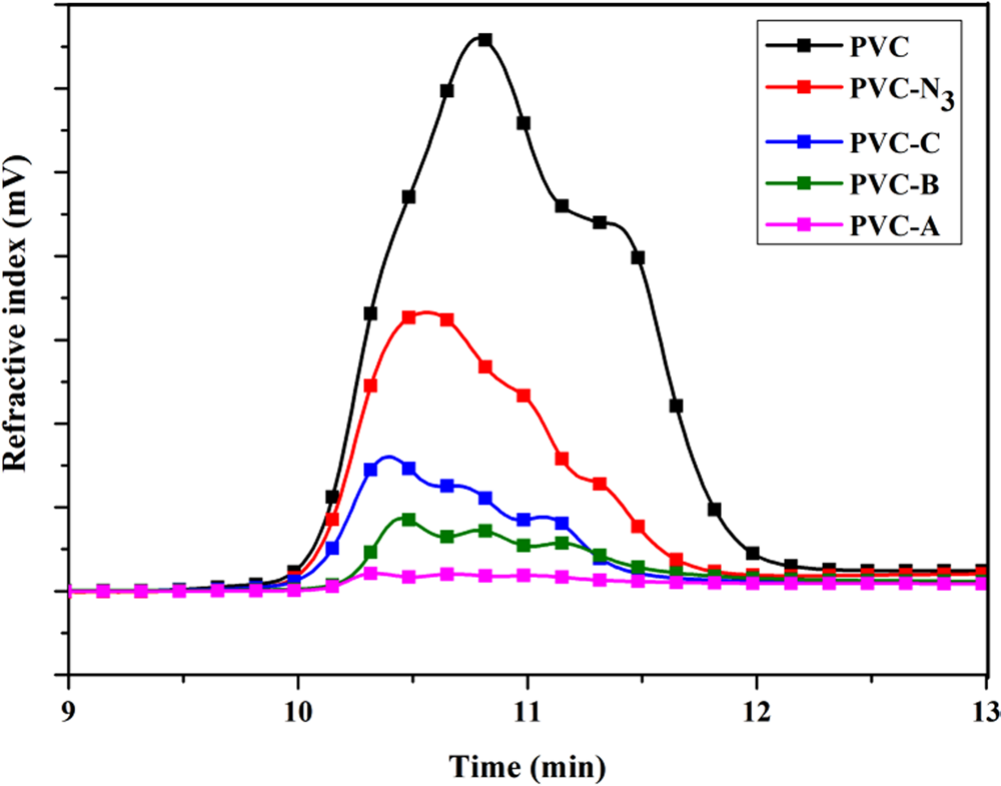
GPC spectra of PVC materials.

Figure 3 shows GPC curves of PVC materials. Table 1 summarizes the GPC data. As seen from Fig. 3 and Table 1, *M*_n_ and *M*_W_ of modified PVC shifted to high molecular weight with more propargyl ether THEIC-MR-P grafted onto the chains of azide functionalized PVC via click chemistry. Peak area of modified PVC shrunk when more propargyl ether THEIC-MR-P was grafted onto the chains of azide functionalized PVC, because macromolecular and high branched polymer (PVC-A) was hard to dissolved in tetrahydrofuran.

**Table 1:** GPC results of modified PVC.

PVC and Modified PVC	Number average relative molecular mass (*M*_n_)	Weight-average molecular weight (*M*_W_)	Polydispersion (*M*_n_/*M*_W_)
PVC	15192	18878	1.2
PVC-N_3_	19123	22769	1.2
PVC-C	21615	24167	1.2
PVC-B	22705	28915	1.3
PVC-A	24087	31059	1.3

### Performances of PVC materials

**Figure 4:**
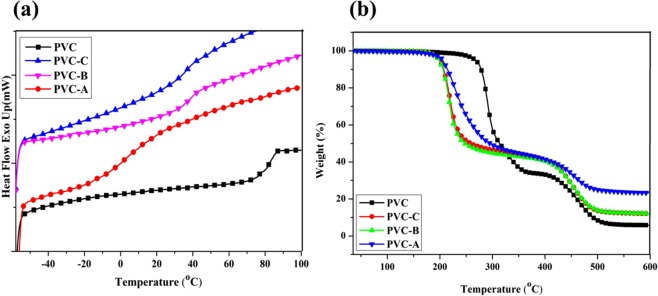
(**a**) DSC curves of modified PVC; (**b**) TGA curves of modified PVC.

Castor oil based derivative grafting onto PVC-N_3_ will decrease glass transition temperature (Tg) of polymer materials by promoting the movement of main chains. In this study, internally plasticized PVC material were obtained by graft copolymerization. Propargyl ether THEIC-MR-P with flexible eater side chain groups will promote the movement of main chains of modified PVC, then decrease the Tg. Figure 4(a) shows the DSC curves. The DSC curves of PVC exhibited an exothermic peak approximately at 87.6 °C, which was corresponded to the Tg of PVC. All the modified PVC materials showed only one melting peak in the DSC curves, which illustrated that all propargyl ether THEIC-MR-P was grafted onto the chains of azide functionalized PVC. Tg of PVC-A, PVC-B and PVC-C was 51.4 °C, 67.1 °C and 76.9 °C, respectively. The results illustrated that Tg of PVC materials reduced when propargyl ether THEIC-MR-P was grafted onto the structure of azide functionalized PVC. Propargyl ether THEIC-MR-P decreased the der Waals’ force which could help to increase segment mobility, and then decreased the Tg of PVC efficiently.

Figure 4(b) shows the TGA curves of PVC materials. All of the PVC materials have two thermal degradation stages. For PVC, dehydrochlorination of PVC occurred at around 200–350 °C and cyclization of the conjugated polyene sequences to form aromatic compounds and char residue occurred at above 350 °C. For internally plasticized PVC(PVC-A, B and C), as seen from Fig. 4(b), which showed that all internally plasticized PVC materials were thermal stable under 200 °C and began to degrade at above 200 °C due to dehydrochlorination of PVC^10^. Internally plasticized PVC materials exhibited less thermal stable with more propargyl ether THEIC-MR-P grafted onto chains of azide functionalized PVC, because more propargyl ether THEIC-MR-P was connected to the PVC-N_3_ matrix, which made the polymer chains loose and easy to move. Thermal degradation of internal plasticizer (propargyl ether THEIC-MR-P) mainly occurred at the around 200–320 °C, which decreased thermal stability of PVC materials^10,20^. Thermal degradation of PVC and propargyl ether THEIC-MR-P plasticizer produced many char residue. Metaphosphorous acid (HPO_2_) was produced from the thermal degradation of propargyl ether THEIC-MR-P, which improved the flame retardancy of PVC materials by promoting to produce more char residue^18,20^. The char residue of PVC, PVC-C, PVC-B and PVC-A was 5.83, 12.04, 12.63 and 20.65%. In fact, the char reside delayed the thermal degradation of modified PVC materials.

**Figure 5:**
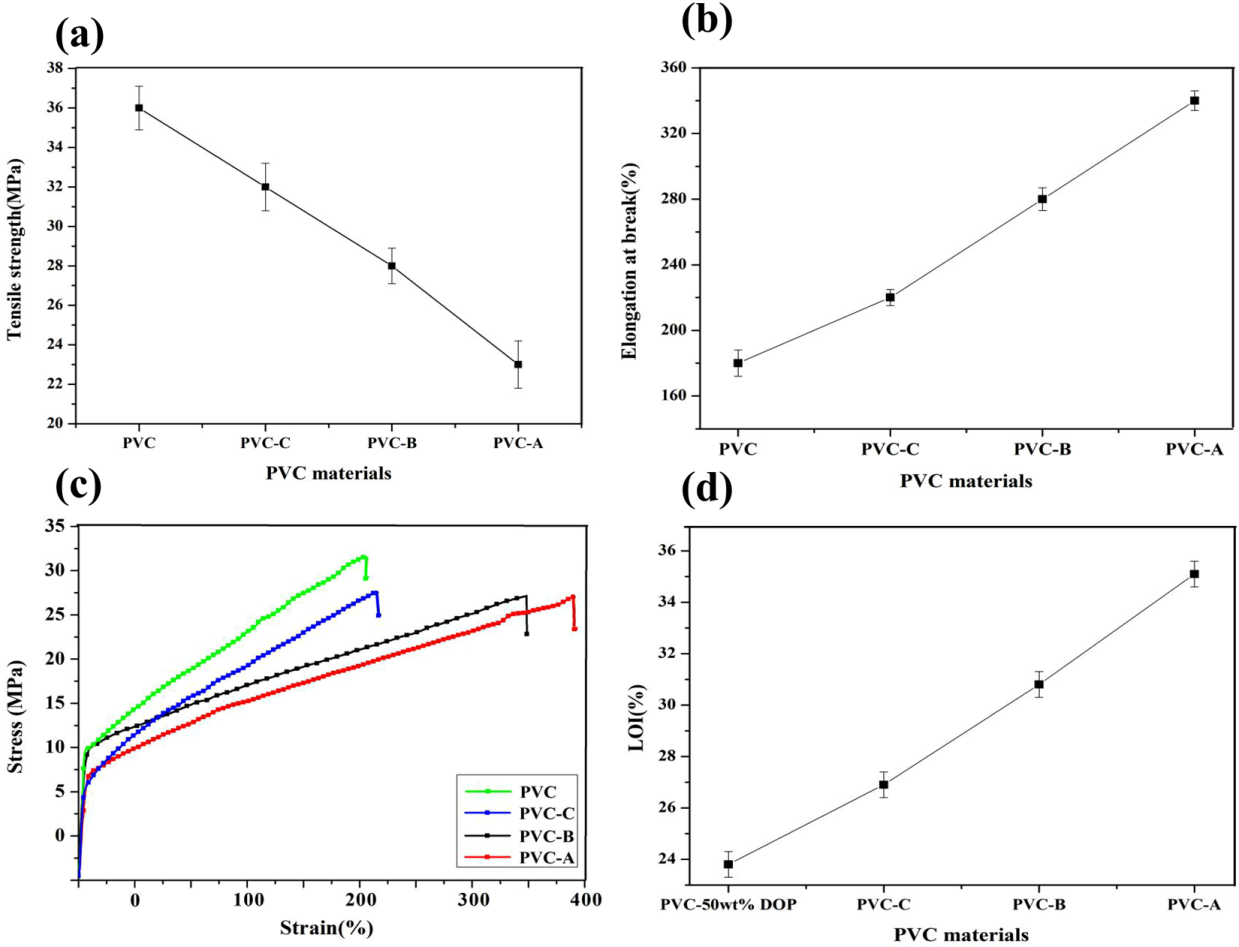
(**a**) Tensile strength of modified PVC. (**b**) Elongation at break of modified PVC. (**c**) Stress-strain curves of modified PVC. (**d**) LOI of modified PVC.

Flexibility of PVC-A, B, C was investigated and compared with PVC. Figure 5(a,b) showed the results. Tensile strength of PVC materials modified with propargyl ether THEIC-MR-P decreased and elongation at break increased gradually with more propargyl ether THEIC-MR-P grafted onto PVC matrix. Tensile strength and elongation at break for PVC was 36.0 MPa and 181.2%. The value of tensile strength and elongation at break for PVC-A reached 22.3 MPa and 326.1% respectively. Figure 5(c) shows the stress-strain curves of PVC materials, the internally plasticized PVC simultaneously exhibit much smaller stress and much larger strain during their deformation compared to PVC, because the propargyl ether THEIC-MR-P, as internal plasticizer, weakened the intermolecular friction forces between the PVC chains. The results illustrated that internally plasticized PVC materials were more flexible than PVC.

In order to study the migration stability of the internal plasticizer, the migration behavior was investigated. The study found that there was not any weight loss for internally plasticized PVC materials after extraction experiments. This illustrated that propargyl ether THEIC-MR-P was grafted onto the polymer matrix. Extraction experiment of plasticizer for PVC plasticized with DOP was studied in our previous studies^11,18^, the results showed that PVC-DOP blends lost plasticizer after the extraction experiments in four different solvents. The use of external plasticizer can’t avoid migration of the PVC products. Therefore, the internal plasticizing method can extend the service life of PVC products.

Flame retardancy of PVC materials were investigated and the limiting oxygen index (LOI) value was showed in Fig. 5(d). The results showed that LOI value of PVC materials containing 50 wt% DOP was 23.6%. LOI value of PVC-C was 26.8%, the value for PVC-A reached 34.7% with more propargyl ether THEIC-MR-P grafted onto PVC chains, which illustrated that flame retardancy of internally plasticized PVC was improved.

## Conclusions

Castor oil derivative containing containing synergistic flame retardant groups of nitrogen and phosphorus was grafted onto the chains of azide functionalized PVC. Chemical structure of the castor oil derivative was investigated. Tg of PVC materials reached 51.4 °C with more propargyl ether THEIC-MR-P grafted on chains of azide functionalized PVC. Thermal stability modified PVC materials is reduced compared with PVC at around 200–320 °C, but more thermal stable at around 320–600 °C. No migration was found after the extraction experiments, which indicated that propargyl ether THEIC-MR-P was covalently linked to the polymer matrix. LOI value of PVC materials modified with castor oil derivative containing THEIC and diethyl phosphate groups reached 34.7%, indicating that the castor oil derivative containing flame retardant groups enhanced flame retardancy of PVC materials. The obtained internally plasticized PVC materials will increase application value of PVC products with high flame retardancy and solvent extraction resistance requirements.

## Methods

### Materials

Castor oil, caustic potash (KOH), anhydrous methanol, tetrabutyl titanate, tetrahydrofuran, 1,3,5-Tris (2-hydroxyethyl) cyanuric acid (THEIC), diethyl chlorophosphate, concentrated sulfuric acid, chloroform, dioctyl phthalate (DOP), propargyl bromide solution, potassium carbonate, sodium azide, N,N-dimethylformamide (DMF), acetone, cuprous bromide, 5,5-dimethyl-2,2-dipyridyl were bought from Nanjing Chemical Reagent Co., Ltd. PVC was bought from Hanwha.

### Synthesis of methyl ricinoleate(MR) and alcoholysis of castor oil methyl with THEIC (THEIC-MR)

Synthesis of methyl ricinoleate(MR) and alcoholysis of castor oil methyl with THEIC (THEIC-MR) was studied in our previous studies^12,18^.

### Synthesis of castor oil derivative containing THEIC(THEIC-MR) and phosphate (THEIC-MR-P)

**Figure 6:**
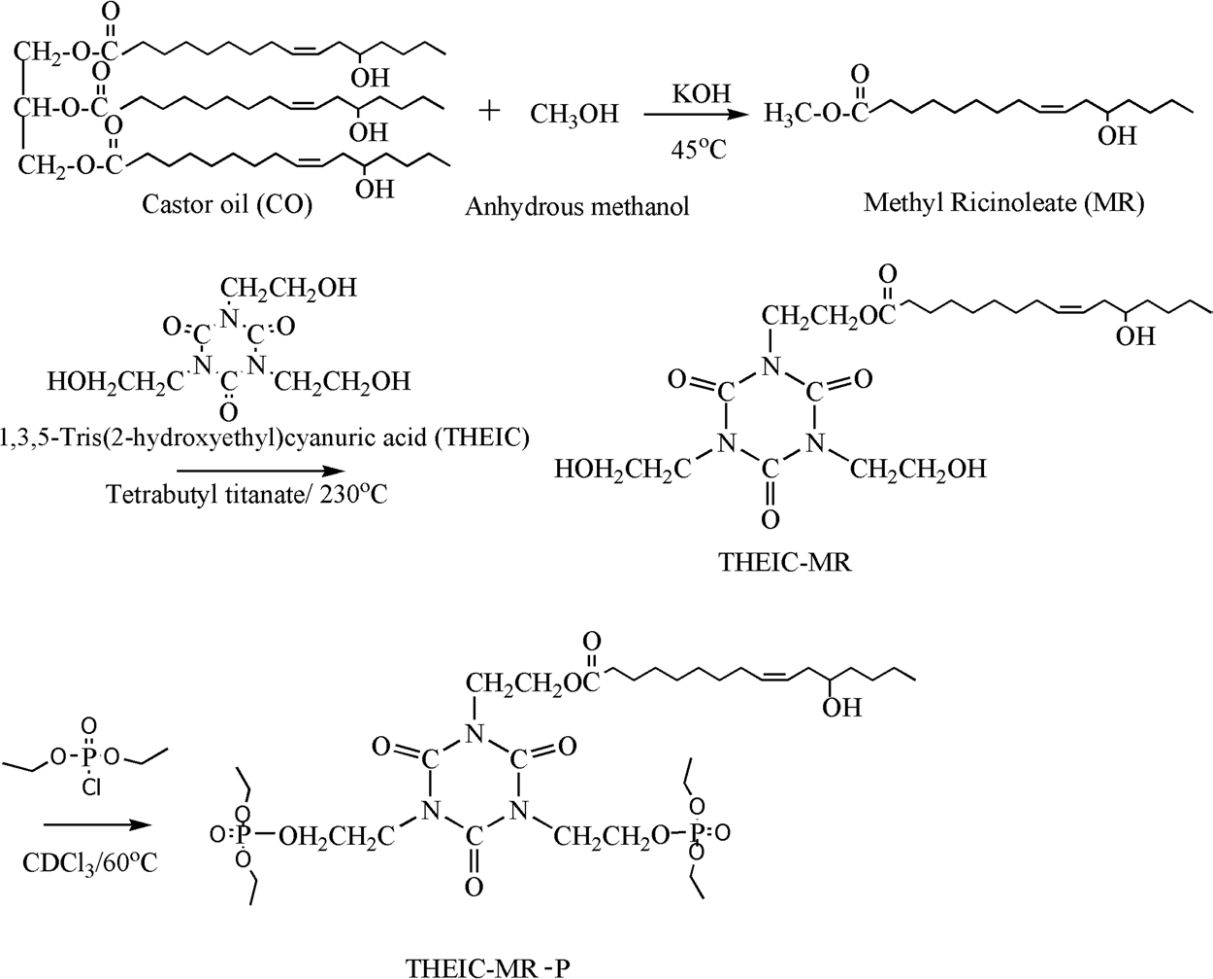
Synthesis of THEIC-MR-P.

THEIC-MR and diethyl chlorophosphate with molar ratio of 1:2 were dissolved in 100 mL of chloroform. The mixture was settled in a 250 mL flask and stirred at 60 °C for 3 h to finish the reaction. THEIC-MR phosphate was gotten after removing chloroform with a rotary. Synthetic route of THEIC-MR-P was presented in Fig. 6.

### Synthesis of Propargyl Ether THEIC-MR-P

THEIC-MR-P (100 mmol), 13.08 g (110 mmol) of propargyl bromide solution and 15.3 g (110 mmol) of potassium carbonate were mixed in 60 mL of acetone, which was stirred at 65 °C for 12 h. The product was obtained after washing with deionized water and purification by evaporating under vacuum. Figure 6 shows the synthesis of propargyl ether THEIC-MR-P.

### Synthesis of azide-functionalized PVC (PVC-N_3_)

PVC-N_3_ was prepared according to our previous studies^19,20^.

### Preparation of modified PVC and films

**Figure 7:**
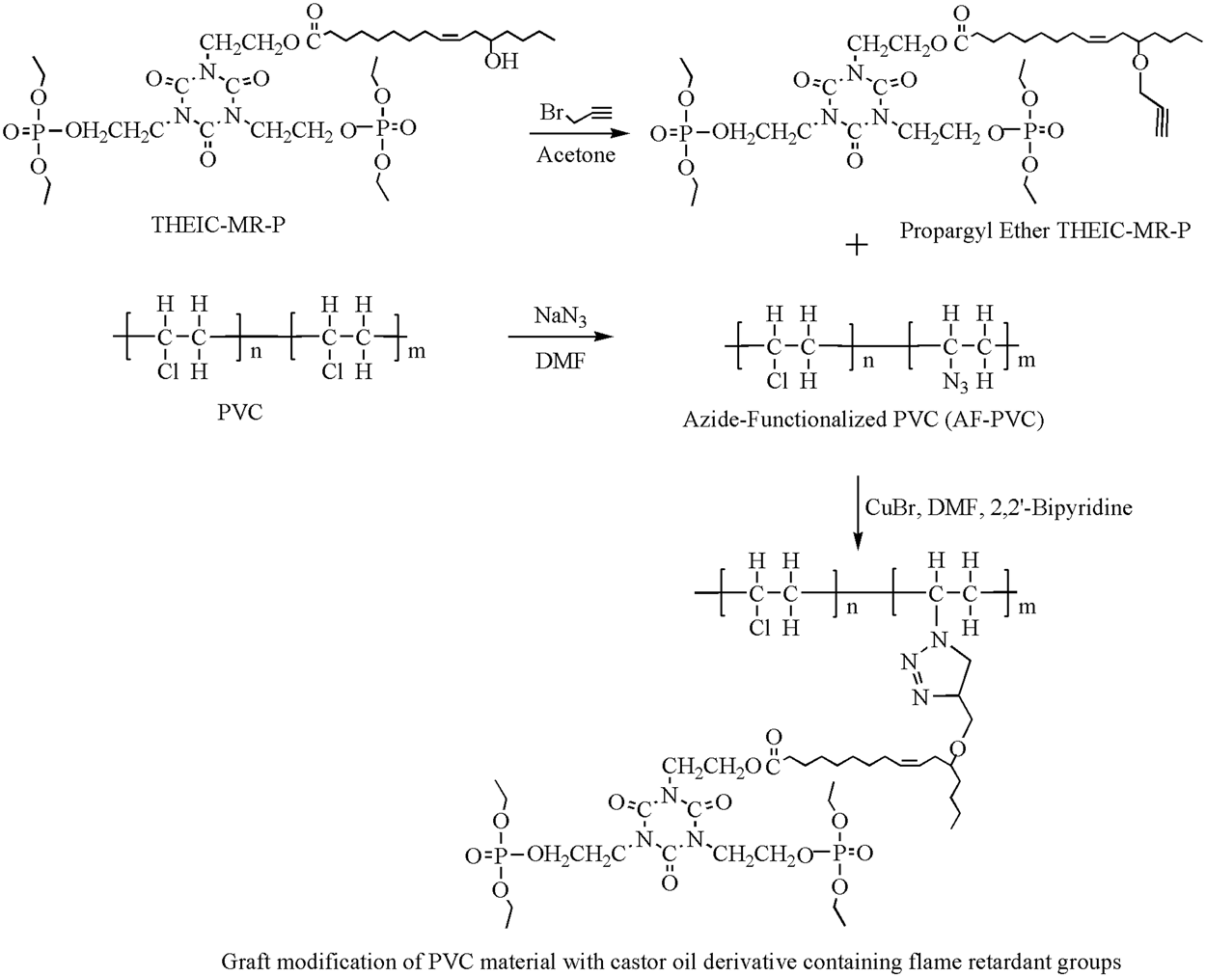
PVC materials modified with THEIC-MR-P.

PVC-A, PVC-B and PVC-C was prepared respectively by dissolving the reactants(as seen from Table 2) in DMF and introduced in round bottom flasks, which was stirred at 30 °C for two days under nitrogen protection. Internally plasticized materials were obtained after removing cuprous bromide, precipitating into water/methanol mixture and drying in a vacuum. The preparation of PVC materials was presented in Fig. 7. PVC and PVC-A, B, C films were prepared via casting method using THF as solvent. Three grams of PVC and PVC-A, B, C was respectively dissolved 50 mL of THF and poured into molds. The polymer films were obtained after removing THF in vacuum drying box at 50 °C for 48 h. PVC films containing 50 wt% of DOP was also prepared using the same method.

**Table 2:** PVC materials and composition of reactants.

PVC materials	PVC-N_3_	Propargyl ether THEIC-MR-P	Cuprous bromide	5,5-dimethyl-2,2-dipyridyl	DMF
PVC-C	2	1.2 g	0.18 g	0.44 g	20 mL
PVC-B	2	0.6 g	0.09	0.22 g	20 mL
PVC-A	2	0.3 g	0.045 g	0.11 g	20 mL

### Characterization

FT-IR analysis was conducted on a Nicolet iS10 FTIR measurement (Thermo Fisher Scientific Inc., USA). Spectra were obtained from 4000 cm^−1^ to 500 cm^−1^. ^1^H NMR spectra of products in deuterated chloroform were detected on an AV-300 measurement. Molecular weight of products in THF was conducted with an Waters GPC measurement. Thermogravimetric analysis (TGA) was detected using a Netzsch TG209F1 measurement in nitrogen atmosphere. Five mg of samples were put into platinum pans and the temperature was increase from room temperature to 600 °C. Tg was characterized using a differential scanning calorimeter(DSC: 200 PC, Netzsch Co., Germany). The samples were detected under a nitrogen atmosphere over the temperature range of −40 to 120 °C. The heating rate was 20 °C/min. Migration stability of PVC materials was tested according to the references^21–23^. The mechanical property of PVC and PVC-A,B,C were investigated with an E43.104 Universal Testing Machine (MTS Instrument Crop., China). Limiting oxygen index (LOI) tests were carried according to the standard of Plastics-Determination of burning behavior by oxygen index (GB/T 2406.1-2008. China) using JF-3 oxygen index measuring instrument (Nanjing Lei instrument, China).
